# A potential role of reward and punishment in the facilitation of the emotion-cognition dichotomy in the Iowa Gambling Task

**DOI:** 10.3389/fpsyg.2013.00944

**Published:** 2013-12-17

**Authors:** Varsha Singh

**Affiliations:** Humanities and Social Science, Indian Institute of TechnologyDelhi, India

**Keywords:** Iowa Gambling Task, instructions, decision making, intertemporality, reward-punishment

## Abstract

The Iowa Gambling Task (IGT) is based on the assumption that a decision maker is equally motivated to seek reward and avoid punishment, and that decision making is governed solely by the intertemporal attribute (i.e., preference for an option that produces an immediate outcome instead of one that yields a delayed outcome is believed to reflect risky decision making and is considered a deficit). It was assumed in the present study that the emotion- and cognition-based processing dichotomy manifests in the IGT as reward and punishment frequency and the intertemporal attribute. It was further proposed that the delineation of emotion- and cognition-based processing is contingent upon reward and punishment as manifested in the frame of the task (variant type) and task motivation (instruction type). The effects of IGT variant type (reward vs. punishment) and instruction type (task motivation induced by instruction types: reward, punishment, reward and punishment, or no hint) on the intertemporal and frequency attributes of IGT decision-making were analyzed. Decision making in the reward variant was equally governed by both attributes, and significantly affected by instruction type, while decision making in the punishment variant was differentially affected by the two attributes and not significantly impacted by instruction type. These results suggest that reward and punishment manifested via task frame as well as the task motivation may facilitate the differentiation of emotion- and cognition-based processing in the IGT.

## Introduction

The Iowa Gambling Task (IGT; Bechara et al., 1994) is widely used to examine the interaction of emotion and cognition in foresighted decision making under conditions of risk and uncertainty. The task tests long-term decision making, and it is believed that inputs from emotion-based processing are beneficial rather than an impediment to long-term decision making (otherwise is believed to be a purely cognition-based process). The IGT offers a choice among four decks of cards, each labeled A′, B′, C′, and D′. The four decks differ in two ways: (a) the net outcome across time (i.e., intertemporal attribute), whereby decks A′ and B′ are poor long-term choices and decks C′ and D′ are safe long-term choices; and (b) the frequency of immediate reward and punishment irrespective of net or long-term outcomes (i.e., frequency attribute), whereby decks A′ and C′ could be perceived as poor choices due to frequent punishments/infrequent rewards and decks B′ and D′ could be perceived as safe choices due to infrequent punishments/frequent rewards.

Task performance was originally believed to depend entirely on the intertemporal attribute (i.e., the choice of immediate outcomes over delayed outcomes is considered disadvantageous), and to disregard frequency of reward and punishment. The reward and punishment schedule of the IGT was assumed to be cognitively impenetrable (i.e., neither the frequency nor the long term payoff/outcome were believed to be cognitively processed), which implied the following: (1) that reward and punishment are indistinguishable from each other and weigh equally, and (2) that decision making is solely driven by the intertemporality of the task choices (i.e., irrespective of reward/punishment, the choice of delayed outcomes over immediate outcomes is considered advantageous). To rule out the sensitivity to reward and punishment as an alternate explanation for myopic decision making in the IGT, Bechara et al. (2000a,b) tested the first implication by comparing intertemporal decision making in two types of IGT variants: the original reward variant (A′B′C′D′) that has “rewards” as a prominent outcome and a punishment variant (E′F′G′H′) that has “loss/punishment” (see Appendix B for variant details) as a prominent outcome. It was demonstrated that decision making was governed by the intertemporal attribute irrespective of the frame or type of IGT variant; in other words, the reward and punishment frame of the IGT variant did not affect intertemporal decision making. However, one study (Maia and McClelland, 2004) of the IGT reward variant showed that participants exhibited knowledge of the reward and punishment schedules (specifically of long term outcome), which indicates these schedules are cognitively penetrable in the IGT reward variant. Moreover, in another study of the IGT reward variant, the frequency of reward and punishment, rather than intertemporality, was found to control decision making (Lin et al., 2007). This evidence negates the assertion that intertemporality is the sole factor governing decision making in the IGT reward variant, and supports a role of reward and punishment in IGT decision making. This influence of reward and punishment on IGT decision making, however, is still largely unclear.

It is assumed in this paper that IGT decisions are based on both frequency of reward and punishment, and intertemporality, and that these two attributes reflect emotion- and cognition-based processing, respectively. It is contended that the role of reward-punishment in the form of IGT variant type and task motivation toward reward and punishment is to differentiate emotion-cognition processing in the IGT. Decision making based on the intertemporal attribute might require the recollection of previous outcomes to determine which decks produced net gains over the trial periods, and therefore might require cognitive resources and involve working memory. On the other hand, decision making based on the frequency attribute imposes no such demand on cognitive resources. Therefore, decision making based on the intertemporal attribute might require cognitive activity, whereas decision making based on the frequency attribute may reflect activity in the emotion-based system. Indeed, Stocco et al. (2009) found a double dissociation in decision making based on both attributes suggesting that intertemporal decision making demands cognitive resources and that the two attributes reflect emotion-cognition dichotomy.

Others have observed that intertemporal decision-making reflects explicit learning (Maia and McClelland, 2004); is dependent on hippocampus-mediated memory systems, such as the declarative memory system (Gupta et al., 2009); and engages working memory (Hinson et al., 2002). Conversely, decision making based on the frequency attribute may reflect automatic processing (Wilder et al., 1998; Stocco et al., 2009), which is indicative of emotion-based processing. Support for this dichotomy comes from dual-process theory of reasoning, which suggests the existence of two systems that process information differently. One system is automatic, emotion-based, and concerned with the present, whereas the second is reflective, cognition-based, and concerned with the future (Tversky and Kahneman, 1971). Therefore, it was assumed in the present study that IGT decision making based on the frequency of reward and punishment reflects automatic emotion-based processing, and decision making based on intertemporality reflects cognitive processing: thus, the two attributes, respectively, reflect emotion- and cognition-based processing. However, it is not yet known which factor determines the dichotomization of emotion-cognition-based processing in the IGT.

In the present study, it is proposed that the frame of the IGT variant and the task motivation toward reward and punishment might influence the differentiation of emotion-cognition-based processing. Contrary to the assumption that intertemporal decision making is not influenced by the frames of the IGT variant (Bechara et al., 2000a), it has been observed that intertemporal decision making is more strengthened in the punishment variant than in the reward variant (e.g., Bechara et al., 2000b, 2002; Must et al., 2006, 2007; Verdejo-Garcia et al., 2006). In one such study, it was observed that the punishment variant, which produces a “loss” outcome for every choice (whereas the reward variant produces a “gain” outcome for every choice), was more conducive to the intertemporal attribute [i.e., cognition-based processing; (Singh and Khan, 2012)]. It was suggested that because the punishment/loss variant triggers risk-seeking while the “reward” variant induces risk-aversion, the punishment variant might require greater cognitive processing than the reward variant. Greater activity in the cognition-based system suggests greater differentiation of emotion-cognition processing in the punishment variant. Therefore, it was expected that the IGT variant type would affect the dichotomization of emotion-cognition-based processing in IGT decision making.

Similar to the assumption that the frame of the IGT variant does not affect intertemporal decision making (Bechara et al., 2000a), the task instructions are also based on an assumption that IGT decision making has equal reward- and punishment-related motivation; the instructions are bidirectional in nature, prompting the decision maker to seek reward as well as avoid punishment. However, contrary to the assumed bi-directionality of task motivation, it has been observed that intertemporal decision making in the IGT is dependent on avoiding punishment rather than seeking reward. For instance, Fernie and Tunney (2006) found that a portion of the instructions that advised the avoidance of “bad” cards was necessary for intertemporal decision making in the reward variant because omission of that portion resulted in poor intertemporal decision making. The omitted part was as follows: “All I can say is that some decks are worse than others. You may find all of them bad, but some are worse than others are. No matter how much you find yourself losing, you can still win if you stay away from the worst decks.” Similarly, Balodis et al. (2006) simplified the instructions by excluding a part that advised subjects to avoid “bad” cards. The simplified instructions were as follows: “In this card game there are four decks of cards. You can draw cards from any of the decks. Every time you click on [*sic*] card, you will win some play-money. With some card draws you will lose money as well. The object of the game is to win as much play-money as possible, or avoid losing as little of the money as possible. You will begin the game with $2000.” These simplified instructions resulted in poor intertemporal decision making, but the reinstatement of the warning resulted in improvement (Balodis et al., 2006). In a previous study, by the present author, it was observed that intertemporal decision making in the IGT reward variant is differentially affected by task motivation toward reward and task motivation toward punishment because a unidirectional version of the standard bidirectional instructions enhanced intertemporal decision making (Singh and Khan, 2012). It has been suggested that the unidirectional instructions (i.e., only to seek reward or to avoid punishment) are less taxing on working memory; this results in more efficient cognition-based processing, and consequently increases intertemporal decision making (Singh and Khan, 2012). According to dual-process theories, efficient cognition-based processing inhibits emotion-based processing (Tversky and Kahneman, 1971; Evans, 2003), and this inhibition may result in more differentiated emotion-cognition based processing. Therefore, in addition to variant type (reward and punishment variant), it was expected that task motivation toward reward and punishment might also affect the differentiation of emotion-cognition processing in the IGT.

Thus, in the present study, it is explored whether varying the reward and punishment frame via variant and/or instruction type affects the emotion-cognition dichotomy, as tested via the two attributes in IGT decision making. It was hypothesized that IGT variant type and task instruction type would influence which attribute governed IGT decision-making.

## Materials and methods

### Sample

Three hundred and twenty healthy undergraduate and graduate students volunteered for the study (mean age = 23.82 years; *SD* = 3.25; male = 160). All participants had more than 18 years of education. Most of the participants were right-handed (86.1%) and non-smokers (93.6%).

### Design

This study used a 2 (reward variant: intertemporal and frequency attributes) × 2 (punishment variant: intertemporal and frequency attributes) × 4 (instruction type: avoid punishment, seek reward, standard, and no hint) design. The two net scores obtained via the two attributes (attribute type) in the two variants (variant type) were the within-subjects variables, and instruction type was the between-subjects variable. The order of variant type presentation was counter-balanced and the sample was gender-balanced; neither presentation order nor gender affected the results (*p* > 0.5).

IGT decision making was analyzed according to the “net score” method (Bechara et al., 1994), in which one total net score was calculated for the five blocks. It is customary to analyze IGT performance using five block-wise net scores rather than one total net score of the five blocks because this method allows for the comparison of participants' learning rate across blocks of trials. However, the focus of the present research at this stage was to differentiate intertemporal decision making (believed to reflect cognition-based processing) from the frequency attribute (believed to reflect emotion-based processing) and to test if the variant type and instruction type affected the differentiation of the two attributes.

To calculate an index of the intertemporal attribute in the reward variant, the number of cards drawn from decks A′ and B′ were added, and their sum was subtracted from the number of cards drawn from decks C′ and D′ ([decks C′ + D′]—[decks A′ + B′]). This was done for a block of 20 trials each, and scores for the five blocks were added to obtain a total net score for the reward variant. The formula used to calculate the intertemporal attribute index in the punishment variant was [“E” + “G”]—[“F” + “H”]. The formula used to calculate the frequency attribute for the reward variant was ([decks “B” + “D”]—[decks “A” + “C”]); for the punishment variant, it was [“F” + “G”]—[“E” + “H”].

### Materials

The computerized IGT progressive reward (A′, B′, C′, D′) and progressive punishment (E′, F′, G′, H′) variants were used. The progressive variant is slightly different from the original IGT in that it exaggerates the future outcome; that is, it increases the magnitude of long-term rewards in the advantageous decks and long-term punishments in the risky decks (Bechara et al., 2000a). Four sets of IGT instructions were used: (1) instructions that prompted the decision maker to seek reward (Reward), (2) instructions that prompted the decision maker to avoid punishment (Punishment), (3) the routinely used bidirectional instructions that prompt the decision maker to seek reward and avoid punishment (Standard), and (4) instructions that contained no prompts toward either reward or punishment (No hint; see Appendix A).

### Procedure

Demographic information was first obtained via questionnaire from each participant. Participants were told that they would be taking part in a decision making experiment where they would be playing/gambling with play-points after which they gave their informed consent. The study was approved by a thesis committee (Research Progress Committee), a departmental committee, and an institute-level committee in charge of overseeing the postgraduate research program. Participants were tested individually in a laboratory and were assigned to one of the experimental conditions. Two IGT variants were presented in a counter-balanced design (i.e., reward variant followed by punishment variant, or vice versa) with one of the four types of instructions (Reward, Punishment, Standard, and No hint). Thus, each participant performed both IGT variants under one type of instruction. Instructions were read before the first variant was presented. After finishing the first variant, a small break was given (5 min). Following this, the same instructions were read for the second variant, and the second variant was presented. When participants had completed both variants, they were debriefed and thanked for their participation in the study.

### Data analysis

Data were analyzed using Statistical Product for Service Solutions version 16 (Chicago, IL, USA). The threshold for statistical significance was set to *p* < 0.05.

## Results

Mean decision making net scores based the two attributes (intertemporal and frequency) in the two variants (reward and punishment) across the four types of instructions (reward, punishment, standard, and no-hint) are presented in Table 1.

**Table 1:** **Descriptive statistics for instruction type, variant type, and attribute type (*n* = 320)**.

**Variant type**	**Attribute type**	**Instruction type**			
		**Reward**	**Punishment**	**Standard**	**No-hint**
Reward	Intertemporal	2.92 (30.78)	10.34 (26.60)	−2.58 (23.88)	−2.78 (22.12)
	Frequency	13.52 (22.07)	16.65 (20.53)	15.80 (26.76)	10.22 (19.20)
Punishment	Intertemporal	19.05 (36.16)	7.65 (30.03)	4.68 (33.92)	9.50 (21.66)
	Frequency	10.18 (29.37)	6.05 (29.47)	7.23 (29.14)	8.65 (29.18)

*Values shown are means and standard deviations (in parentheses)*.

**Figure 1:**
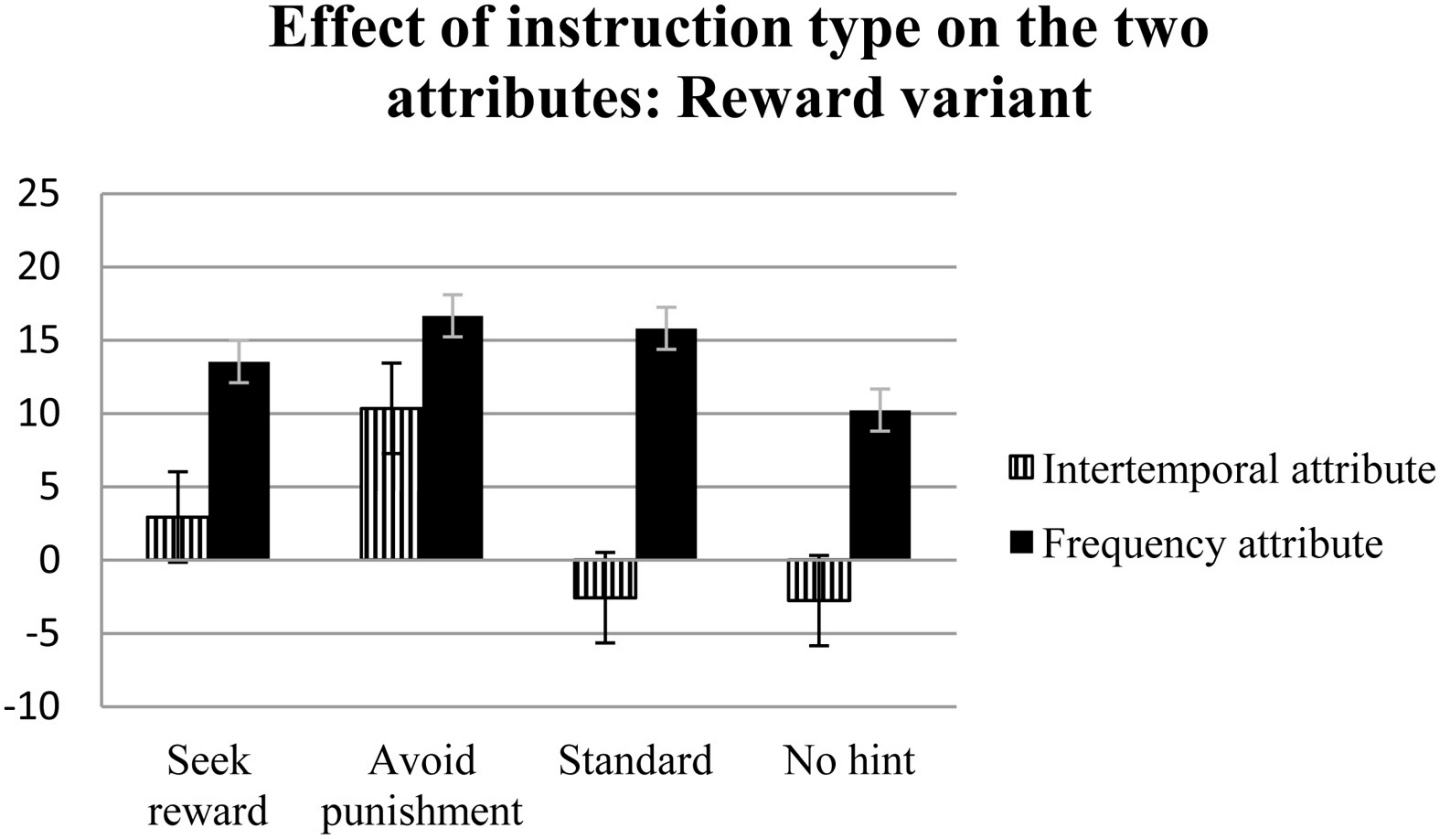
**The effects of four types of instructions on decision making in the reward variant, assessed via two attributes: intertemporal (C′ + D′)—(A′ + B′) and frequency (B′ + D′)—(A′ + C′)**. Error bars represent standard errors.

**Figure 2:**
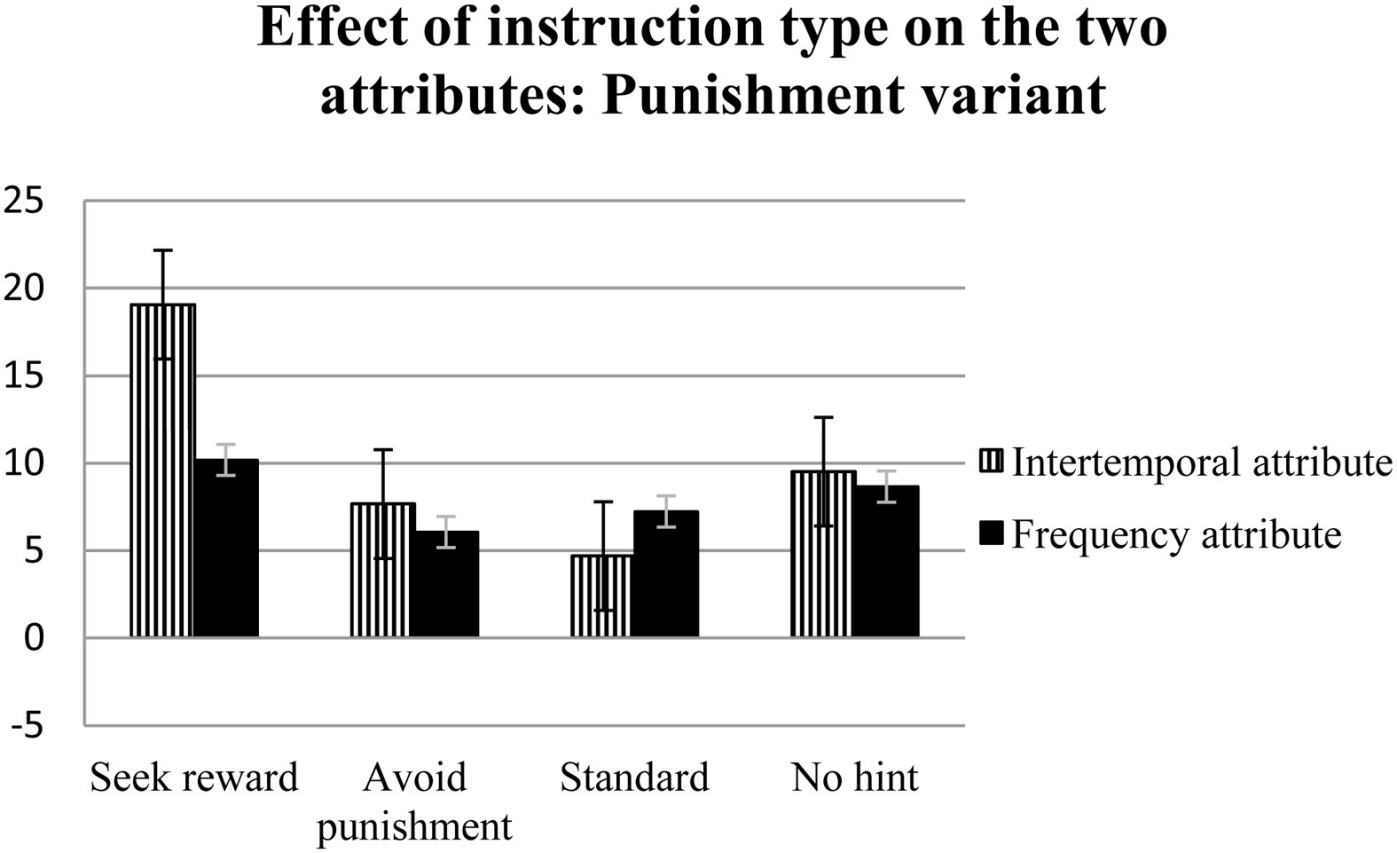
**The effects of four types of instructions on decision making in the punishment variant, assessed via two attributes: intertemporal (E′ + G′)—(F′ + H′) and frequency (F′ + G′)—(E′ + H′)**. Error bars represent standard errors.

The results of a repeated-measures analysis of variance using the four net scores (obtained on the basis of the two attributes in the two variants) showed a non-significant main effect of attribute type and a significant interaction of instruction and attribute type for the reward variant [*F*_(3, 312)_ = 4.52, η^2^_*p*_ = 0.04, *p* < 0.01] (see Figure 1). Multiple comparisons for the reward variant using Tukey's Honestly Significant Difference test showed that only the unidirectional instructions for seeking reward differed from the standard and no hint instructions; however, the significance levels of these variables were *p* = 0.08 and *p* = 0.09, respectively, indicating marginal significance. A significant main effect of attribute type [*F*_(1, 312)_ = 9.36, η_*p*2_ = 0.03, *p* < 0.01], but no interaction effect of instruction and attribute type, was observed for the punishment variant (see Figure 2).

## Discussion

The study examined the effects of task motivation and IGT variant framing on the two attributes of decision making in the IGT. The results indicated that decision-making was governed equally by both attributes, and that task instructions affected attribute type in the reward variant. In the punishment variant, decision-making was differentially governed by the two attributes, and the task instructions did not affect the attributes.

These results are consistent with previous studies that showed that decision making in the reward variant is not solely based on the intertemporal attribute (e.g., Chiu and Lin, 2007; Lin et al., 2007; Chiu et al., 2008), which suggests the influence of more than one attribute on decision making in the reward variant. The present results showed an interaction between task instructions and attribute type in the reward variant, which is consistent with the observation that task instructions—specifically those that advise subjects to avoid “bad” cards—are critical for intertemporal decision making in the reward variant (e.g., Blair and Cipolotti, 2000; Balodis et al., 2006; Fernie and Tunney, 2006). The results suggest that the bifurcation of task motivation toward reward and punishment might be differentially conducive to the two attributes (i.e., it facilitates cognitive or emotional processing), and that it might facilitate dichotomization of the emotion-cognition processing when the IGT is framed in a reward variant.

The differential governing of decision making by the two attributes in the punishment variant suggests a dominance of one attribute. This observation is consistent with previous claims that intertemporal decision making dominates in the punishment variant (e.g., Bechara et al., 2000b, 2002; Must et al., 2006, 2007; Verdejo-Garcia et al., 2006). Therefore, the punishment variant may be more effective than the reward variant at differentiating between emotion- and cognition-based decision making. When the two attributes are well-differentiated, task instructions do not seem to play a critical role. The results further corroborate the assertion that instruction-induced task motivation toward reward and punishment plays a role in the dichotomization of emotion-cognition processing. Results additionally show that task motivation differentially affects decision making in the reward and punishment variants of the IGT. Instructions play an important role in the reward variant, where there is equivocal attribute preference (i.e., undifferentiated emotion-cognition based processing), but not in the punishment variant, where there is unequal attribute preference (i.e., differentiated emotion-cognition based processing).

Furthermore, the present results support the earlier observation that bifurcating task instructions into reward-seeking and punishment-avoidance might reduce working memory demands (Singh and Khan, 2012), resulting in more efficient cognition-based processing and inhibition of emotion-based processing (i.e., facilitation of the differentiation between the two attributes), in other words, a well-differentiated emotion-cognition based processing. This explanation (about the role of working memory in improving cognition-based processing via a well-differentiation of emotion-cognition based processing) is consistent with earlier findings that intertemporal decision making in the IGT is dependent on working memory. For instance, studies have reported that performing a secondary task interfered with working memory and negatively affected intertemporal decision making in the IGT reward variant (Turnbull et al., 2005; Stocco et al., 2009). This implies that one of the ways to rectify intertemporal decision-making impairments, which are synonymous with decision making deficits in a clinical sample (e.g., substance abuse), might be to try and dissociate reward-seeking motivation from punishment-avoidance motivation through the utilization of unidirectional instructions. Impaired intertemporal decision making is believed to be due to a failure to integrate both emotion and cognition-based processing (e.g., Killgore et al., 2007). An interesting but preliminary theoretical implication of the present results in this regard, which requires further investigation, is the possibility that dissociating rather than integrating emotion-cognition processing might result in better intertemporal decision making in the IGT.

Future studies that examine why the punishment frame of the IGT engages cognition-based processing and consequently facilitates the differentiation of emotion- and cognition-based processing to a greater degree than does the reward frame would be informative. The speculation that the reward and punishment frames of the IGT differentially rely on emotion- and cognition-based processing is consistent with the results of at least one study. In this experiment, the Task of Cups in a reward and punishment frame was used to analyze decision making in patients with a lesion in the amygdala, a brain region that mediates emotional responsivity (Weller et al., 2007). It was observed that participant's decision making was impaired in the reward frame and intact in the punishment frame, suggesting that decision making in the punishment frame might not rely as much on emotion-based processing as does decision making in the reward frame. This supports the present assertion that the loss frame in the IGT might engage cognition-based processing to a greater extent than the reward frame, thus resulting in a more pronounced dichotomy of emotion-cognition based processing in the loss frame compared with the reward frame.

One limitation of the present study is the lack of accounting for differences in personality (Franken and Muris, 2005) and mood (Suhr and Tsanadis, 2007), which may have affected IGT decision-making. The absence of a real-money reward or a material incentive for participation might be another limitation; however, at least one study has shown that there is no difference in IGT decision making based on whether incentives are real (monetary) or facsimiles (Bowman and Turnbull, 2003). Nevertheless, these limitations should be taken into account when interpreting the findings of this study. The findings of the present study suggest that reward and punishment manipulated via IGT task frame and task motivation play a critical role in IGT decision making, and that role might include the delineation of emotion- and cognition-based processing.

### Conflict of interest statement

The author declares that the research was conducted in the absence of any commercial or financial relationships that could be construed as a potential conflict of interest.

## Figures and Tables

**Figure A1:**
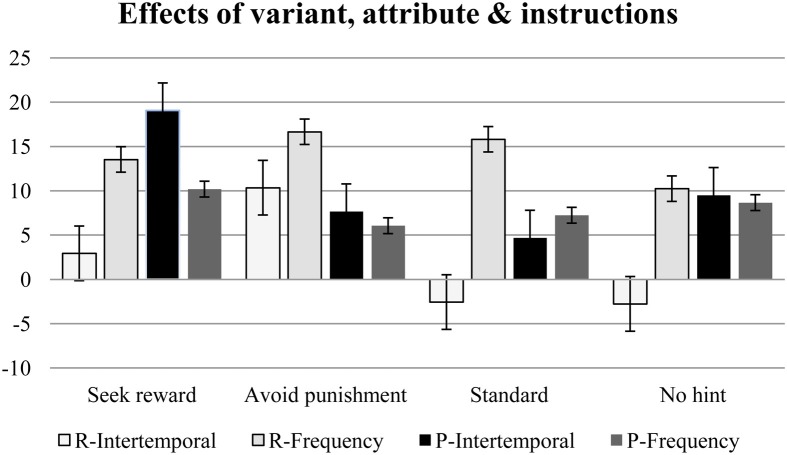
**The effects of four types of instructions on decision making in the reward variant were assessed via two attributes: intertemporal (C′ + D′)—(A′ + B′) and frequency (B′ + D′)—(A′ + C′); and the punishment variant was assessed via two attributes: intertemporal (E′ + G′)—(F′ + H′) and frequency (F′ + G′)—(E′ + H′)**. Error bars represent standard errors.

## Appendix A

Four types of instructions were used in the study: standard (1a and 1b), seek reward (2), avoid punishment (3), and no hint (4a and 4b) instructions.

(1a) Standard instructions, reward variant: “In front of you on the screen, there are four decks of cards: A′, B′, C′, and D′. When we begin the game, I want you to select one card at a time by clicking on a card from any deck. Each time you select a card, the computer will tell you that you won some money. I don't know how much money you will win. You will find this out as you go along. Every time you win, the green bar at the top of the screen gets bigger. Every so often, when you click on a card, the computer will tell you that you won some money as usual, but it will also say that you lost some money as well. I don't know when you will lose or by how much. You will find out as you go along. Every time you lose, the green bar at the top of the screen gets smaller. You are absolutely free to switch from one deck to another at any time, and as often as you wish. The goal of the game is to win as much money as possible and to avoid losing as much money as possible. You won't know when the game will end. Simply keep on playing until the computer stops. You will have $2000 of credit, shown by the green bar, at the start of the game. The only hint I can give you, which is the most important thing to note, is this: Out of these four decks of cards, some are worse than others. To win, you should try to stay away from bad decks. No matter how much you find yourself losing, you can still win the game if you avoid the bad decks. Moreover, the computer does not change the position of the decks once the game begins. It does not make you lose at random, or make you lose money based on the last card you picked.”
(1b) Standard instructions, punishment variant: “In front of you on the screen, there are four decks of cards: E′, F′, G′, and H′. When we begin the game, I want you to select one card at a time by clicking on a card from any deck. Each time you select a card, the computer will tell you that you lost some money. I don't know how much money you will lose. You will find this out as you go along. Every time you lose, the green bar at the top of the screen gets smaller. Every so often, when you click on a card, the computer will tell you that you lost some money as usual, but it will say that you gained some money as well. I don't know when you will gain or by how much. You will find out as you go along. Every time you gain some money, the green bar at the top of the screen gets bigger. You are absolutely free to switch from one deck to the other at any time, and as often as you wish. The goal of the game is to avoid losing as much money as possible and to win as much money as possible. You won't know when the game will end. Simply keep on playing until the computer stops. You will have $2000 of credit, shown by the green bar, at the start of the game. The only hint I can give you, which is the most important thing to note, is this: Out of these four decks of cards, some are better than others. To win, you should try to choose from the good decks. No matter how much you find yourself losing, you can still win the game if you choose from the good decks. Moreover, the computer does not change the position of the decks once the game begins. It does not make you win or lose at random, or make you win or lose money based on the last card you picked.”
(2) Seek reward instructions: Same as in the standard instructions, reward variant, except that the bold text is now “The goal of the game is to win as much money as possible.”
(3) Avoid punishment instructions: Same as in the standard instructions, punishment variant, except that the bold text is now “The goal of the game is to avoid losing as much money as possible.”
(4a) No hint instructions, reward variant: “In front of you on the screen, there are four decks of cards: A′, B′, C′, and D′. When we begin the game, I want you to select one card at a time by clicking on a card from any of these decks. Sometimes you will win points, and sometimes you will lose points. You are absolutely free to switch from one deck to another at any time, and as often as you wish. You won't know when the game will end. Simply keep on playing until the computer stops. You will have $2000 of credit, shown by the green bar, at the start of the game. Moreover, the computer does not change the position of the decks once the game begins. It does not make you lose at random, or make you lose money based on the last card you picked.”
(4b) No hint instructions, punishment variant: “In front of you on the screen, there are four decks of cards: E′, F′, G′, and H′. When we begin the game, I want you to select one card at a time by clicking on a card from any of these decks. Sometimes you will win points and sometimes you will lose points. You are absolutely free to switch from one deck to the other at any time, and as often as you wish. You won't know when the game will end. Simply keep on playing until the computer stops. You will have $2000 of credit, shown by the green bar, at the start of the game. Moreover, the computer does not change the position of the decks once the game begins. It does not make you lose at random, or make you lose money based on the last card you picked.”

## Appendix B

1. Deck information in the two IGT variants: The IGT reward variant offers a choice among four decks of cards labeled A′, B′, C′, and D′. Unlike the original paper-and-pencil based task (ABCD), the computerized task (A′B′C′D′) has increased delayed punishment and therefore amplifies the effect of disadvantageous choices (see Bechara et al., 2000a, for differences between the original and the computerized version). Unbeknownst to the decision maker, decks A′ and B′ have high immediate rewards (100 points per card-pick) but 50% of cards drawn from deck A′ giving a loss of 35–100 points and 10% of cards drawn from deck B′ giving a loss of 250 points, such that 10 cards drawn from decks A′ and B′ result in a net loss of 250 points. Decks C′ and D′ have small immediate rewards (50 points per card-pick) with 50% of cards drawn from deck C′ giving a loss of 25–75 points and 10% of cards drawn from deck D′ giving a loss of 250 points, such that 10 cards drawn from decks C′ and D′ result in a net gain of 250 points. The punishment variant offers a choice between four decks of cards labeled E′, F′, G′, and H′. After a card is picked, the “loss” is announced, which at times is followed by a “gain.” Decks F′ and H′ give immediate low losses and a low net gain, while decks E′ and G′ give immediate high losses and a high net gain. Long-term advantageous decision making is reflected in choosing high-immediate-loss decks (decks E′ and G′) and avoiding low-immediate-punishment decks. Although both variants offer both rewards and punishments, the prominent outcome in the reward variant is a “win,” while that in the punishment variant is a “loss,” which underlies the assertion that a positive frame (i.e., “gain”) is triggered in the reward variant and a negative frame (i.e., “loss”) is triggered in the punishment variant.
2. The graph shows the effects of variant, instruction, and attribute type in IGT decision-making (Figure A1).
